# 3-Hydr­oxy-3-[(2-methyl­propano­yl)meth­yl]indolin-2-one

**DOI:** 10.1107/S1600536809024611

**Published:** 2009-07-01

**Authors:** Gang Chen, Bin Liu, Ying Tang, Jingfang Xu

**Affiliations:** a College of Chemistry and Chemical Engineering, Xi’an Shiyou University, Dianzi’er Road No. 18 Xi’an 710065, Xi’an, People’s Republic of China; b College of Environment and Chemical Engineering, Xi’an Polytechnic University, Nouth Jinhua Roud No. 19 Xi’an 710048, Xi’an, People’s Republic of China

## Abstract

The title compound, C_13_H_15_NO_3_, was synthesized by the Aldol reaction of isatin and 3-methyl­butan-2-one refluxing in methanol catalyzed by dimethyl­amine. The packing of the mol­ecules in the crystal structure features inter­molecular N—H⋯O and O—H⋯O hydrogen bonds.

## Related literature

For related structures, see: Garden *et al.* (2002 ▶); Li, *et al.* (2008 ▶). For the bioactivity of derivatives, see: Glover *et al.* (1988 ▶); Marti & Carreira (2003 ▶); Pandeya *et al.* (2000 ▶); Sun *et al.* (1998 ▶); Teitz *et al.* (1994 ▶).
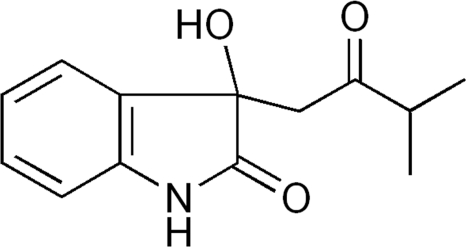


## Experimental

### 

#### Crystal data

C_13_H_15_NO_3_
*M* *_r_* = 233.26
Monoclinic, 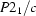

*a* = 11.885 (2) Å
*b* = 5.9244 (12) Å
*c* = 16.695 (3) Å
β = 98.60 (3)°
*V* = 1162.3 (5) Å^3^
*Z* = 4
Mo *K*α radiation
μ = 0.10 mm^−1^
*T* = 293 K
0.23 × 0.18 × 0.15 mm

#### Data collection

Bruker SMART CCD area-detector diffractometer
Absorption correction: multi-scan (*SADABS*; Sheldrick, 2005 ▶) *T*_min_ = 0.945, *T*_max_ = 0.985
8623 measured reflections
2577 independent reflections
1902 reflections with *I* > 2σ(*I*)
*R*_int_ = 0.028

#### Refinement

*R*[*F* ^2^ > 2σ(*F* ^2^)] = 0.045
*wR*(*F* ^2^) = 0.161
*S* = 1.09
2577 reflections
155 parameters
H-atom parameters constrained
Δρ_max_ = 0.24 e Å^−3^
Δρ_min_ = −0.22 e Å^−3^

### 

Data collection: *SMART* (Bruker, 2002 ▶); cell refinement: *SAINT* (Bruker, 2002 ▶); data reduction: *SAINT*; program(s) used to solve structure: *SHELXS97* (Sheldrick, 2008 ▶); program(s) used to refine structure: *SHELXL97* (Sheldrick, 2008 ▶); molecular graphics: *ORTEP-3* (Farrugia, 1997 ▶) and *DIAMOND* (Brandenburg, 1999 ▶); software used to prepare material for publication: *WinGX* (Farrugia, 1999 ▶).

## Supplementary Material

Crystal structure: contains datablocks I, global. DOI: 10.1107/S1600536809024611/ww2134sup1.cif

Structure factors: contains datablocks I. DOI: 10.1107/S1600536809024611/ww2134Isup2.hkl

Additional supplementary materials:  crystallographic information; 3D view; checkCIF report

## Figures and Tables

**Table 1:** Hydrogen-bond geometry (Å, °)

*D*—H⋯*A*	*D*—H	H⋯*A*	*D*⋯*A*	*D*—H⋯*A*
N1—H1*A*⋯O2^i^	0.86	2.22	3.0049 (18)	151
O2—H2*A*⋯O1^ii^	0.82	2.00	2.8220 (17)	175

Symmetry codes: (i) 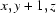; (ii) 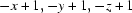.

**Fig. 1.**
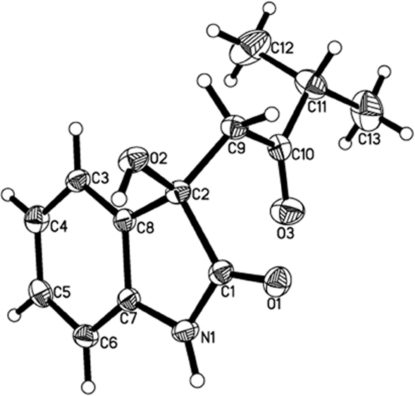
An ORTEP-3 drawing of compound I, with the atom-numbering scheme and 30% probability displacement ellipsoids.

**Fig. 2.**
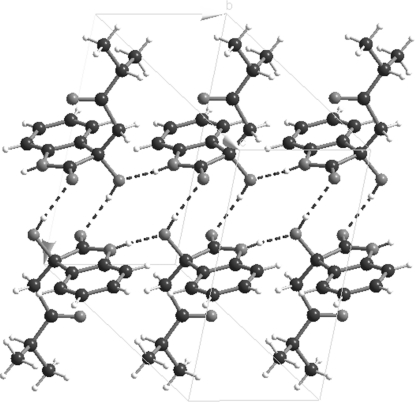
Packing diagram of the title compound. Dashed lines indicate hydrogen bonds.

## supplementary crystallographic information

### Comment

Isatin, an endogenous compound in mammalian tissues and body fluids (Glover, *et al.*, 1988), has caught great attention of many researchers as a versatile lead molecule for designing of potential drugs (Pandeya, *et al.*, 2000; Sun, *et al.*, 1998; Teitz, *et al.*, 1994). In our previous study, 3-hydroxy-3-(2-oxo-propyl)-1,3-dihydro-indol-2-one, yielded from the Aldol reaction of isatin with acetone, was found to be a new chemical activator against tobacco mosaic virus (TMV) infection (Li, 2008). In tobacco plant, external application of 3-hydroxy-3-(2-oxo-propyl)-1,3-dihydro-indol-2-one results in restriction of TMV multiplication and spread, accumulation of salicylic acid level expression of PR-1 gene, and activation and increase of phenylalanine ammonia-lyase (PAL) activity. With these findings, some analogs need to be synthesized for structure activity relationship research to find more potent molecules.

One of these analogs, 3-hydroxy-3-(3-methyl-2-oxo-butyl)-1,3-dihydro-indol-2-one (compound I), was synthesized by the Aldol reaction of isatin with 3-methyl-butan-2-one. In order to provide the structural information of compound I, we studied its crystal structure. The title compound was synthesized in a one step Aldol reaction of isatin (0.01 mmol) with 3-methyl-butan-2-one, according to the reported method (Garden *et al.*, 2002). The molar ratio of isatin: 3-methyl-butan-2-one = 1:3 refluxed in methanol gave compound I in 72% yield, and colorless crystals of compound I were obtained in ethanol by recrystallization. The values of the geometric parameters of compound I are within normal ranges and experimental errors.

The molecular structure of compound I is illustrated in Fig. 1. In the molecule, the 2-oxo-indole ring is planar, and the angle between hydroxyl group and 3-methyl-2-oxo-butyl group is 105.10 (12)°. The intermolecular interactions are primarily responsible for the formation of the crystal structure of compound I. Each molecule is fixed by four hydrogen bond of other three molecules. N1–H1 acts as a hydrogen bond donor, and O1 is a hydrogen bond accepter. To O2–H2, it acts as both a hydrogen bond donor and a hydrogen bond accepter, which connects with two molecules by O–H···O and O···H–N with the angle of 119.21 (9)°. There is no anticipated intramolecular hydrogen bond between O2–H2 and O3, and O3 is not involved in any hydrogen bond (Fig. 2).

### Experimental

A mixture of isatin (0.01 mmol) and 3-methyl-butan-2-one (0.03 mmol) was refluxed in methanol (60 ml), catalyzed by a drop of dimethylamine, until the disappearance of the starting material, as evidenced by thin-layer chromatography. The solvent was removed *in vacuo* and the residue was separated by column chromatography (silica gel, petroleum ether/ethyl acetate = 5:1), giving the title compound I 0.168 g, yield 72%. ^1^H-NMR (CDCl_3_, 400 MHz): 8.5 (1*H*,s), 7.32 (1*H*, d, J = 7.2 Hz), 7.24 (1*H*, t, J = 7.6 Hz), 7.03 (1*H*, t, J = 7.2 Hz), 6.88 (1*H*, d, J = 16.8 Hz), 4.81 (1*H*, s), 3.25 (1*H*, d, J = 16.8 Hz), 3.17 (1*H*, d, J = 16.8 Hz), 2.56 (1*H*, q, J = 6.8 Hz), 1.05 (6*H*, dd, J = 29.2, 6.8 Hz); ^13^C-NMR (CDCl_3_, 100 MHz): 213.9, 178.5, 140.6, 130.3, 129.9, 124.1, 123.1, 110.5, 74.7, 45.6, 41.9, 17.6; MS (EI) m/*z*: 219 (*M*^+^). 30 mg of compound I was dissolved in 30 ml methanol and the solution was kept at room temperature for 4 d, natural evaporation gave colorless single crystals of compound I suitable for X-ray analysis.

### Refinement

All H atoms were positioned geometrically, with C–H = 0.93–0.98 Å, and refined using riding model, with *U*_iso_(H) = 1.2*U*_eq_(carrier).

### Crystal data

**Table:**

C_13_H_15_NO_3_	*F*(000) = 496
*M**_r_* = 233.26	*D*_x_ = 1.333 Mg m^−^^3^
Monoclinic, *P*2_1_/*c*	Mo *K*α radiation, λ = 0.71073 Å
Hall symbol: -P 2ybc	Cell parameters from 3113 reflections
*a* = 11.885 (2) Å	θ = 1.9–27.4°
*b* = 5.9244 (12) Å	µ = 0.10 mm^−^^1^
*c* = 16.695 (3) Å	*T* = 293 K
β = 98.60 (3)°	Block, colorless
*V* = 1162.3 (5) Å^3^	0.23 × 0.18 × 0.15 mm
*Z* = 4	

### Data collection

**Table:**

Bruker SMART CCD area-detector diffractometer	2577 independent reflections
Radiation source: fine-focus sealed tube	1902 reflections with *I* > 2σ(*I*)
graphite	*R*_int_ = 0.028
φ and ω scans	θ_max_ = 27.5°, θ_min_ = 1.7°
Absorption correction: multi-scan (*SADABS*; Sheldrick, 2005)	*h* = −15→14
*T*_min_ = 0.945, *T*_max_ = 0.985	*k* = −7→7
8623 measured reflections	*l* = −21→15

### Refinement

**Table:**

Refinement on *F*^2^	Secondary atom site location: difference Fourier map
Least-squares matrix: full	Hydrogen site location: inferred from neighbouring sites
*R*[*F*^2^ > 2σ(*F*^2^)] = 0.045	H-atom parameters constrained
*wR*(*F*^2^) = 0.161	*w* = 1/[σ^2^(*F*_o_^2^) + (0.0562*P*)^2^ + 0.2707*P*] where *P* = (*F*_o_^2^ + 2*F*_c_^2^)/3
*S* = 1.09	(Δ/σ)_max_ < 0.001
2577 reflections	Δρ_max_ = 0.24 e Å^−^^3^
155 parameters	Δρ_min_ = −0.22 e Å^−^^3^
0 restraints	Extinction correction: *SHELXL*, Fc^*^=kFc[1+0.001xFc^2^λ^3^/sin(2θ)]^-1/4^'
Primary atom site location: structure-invariant direct methods	Extinction coefficient: 0.018 (4)

### Special details

**Table:**

Geometry. All e.s.d.'s (except the e.s.d. in the dihedral angle between two l.s. planes) are estimated using the full covariance matrix. The cell e.s.d.'s are taken into account individually in the estimation of e.s.d.'s in distances, angles and torsion angles; correlations between e.s.d.'s in cell parameters are only used when they are defined by crystal symmetry. An approximate (isotropic) treatment of cell e.s.d.'s is used for estimating e.s.d.'s involving l.s. planes.
Refinement. Refinement of *F*^2^ against ALL reflections. The weighted *R*-factor *wR* and goodness of fit *S* are based on *F*^2^, conventional *R*-factors *R* are based on *F*, with *F* set to zero for negative *F*^2^. The threshold expression of *F*^2^ > σ(*F*^2^) is used only for calculating *R*-factors(gt) *etc*. and is not relevant to the choice of reflections for refinement. *R*-factors based on *F*^2^ are statistically about twice as large as those based on *F*, and *R*- factors based on ALL data will be even larger.

### Fractional atomic coordinates and isotropic or equivalent isotropic displacement parameters (Å^2^)

**Table:**

	*x*	*y*	*z*	*U*_iso_*/*U*_eq_	
O2	0.57929 (9)	0.14669 (16)	0.55228 (6)	0.0335 (3)	
H2A	0.5183	0.2128	0.5411	0.050*	
O1	0.62325 (9)	0.59992 (19)	0.48205 (6)	0.0368 (3)	
N1	0.60709 (11)	0.6720 (2)	0.61556 (8)	0.0324 (3)	
H1A	0.5830	0.8088	0.6103	0.039*	
O3	0.87069 (11)	0.5445 (2)	0.61665 (9)	0.0542 (4)	
C1	0.63221 (12)	0.5435 (2)	0.55343 (9)	0.0292 (4)	
C8	0.66420 (12)	0.3373 (2)	0.67659 (9)	0.0278 (4)	
C9	0.77543 (13)	0.2149 (3)	0.56337 (10)	0.0320 (4)	
H9A	0.7868	0.0611	0.5829	0.038*	
H9B	0.7679	0.2105	0.5047	0.038*	
C10	0.87990 (14)	0.3519 (3)	0.59554 (10)	0.0347 (4)	
C7	0.62536 (13)	0.5539 (2)	0.69000 (9)	0.0289 (4)	
C2	0.66509 (12)	0.3034 (2)	0.58702 (9)	0.0276 (4)	
C3	0.69101 (13)	0.1899 (3)	0.74041 (9)	0.0322 (4)	
H3A	0.7185	0.0462	0.7320	0.039*	
C6	0.61006 (14)	0.6260 (3)	0.76617 (10)	0.0351 (4)	
H6A	0.5832	0.7702	0.7746	0.042*	
C4	0.67614 (14)	0.2602 (3)	0.81790 (10)	0.0383 (4)	
H4A	0.6931	0.1621	0.8615	0.046*	
C5	0.63660 (14)	0.4736 (3)	0.83018 (10)	0.0381 (4)	
H5A	0.6273	0.5176	0.8823	0.046*	
C11	0.99369 (15)	0.2365 (3)	0.59805 (12)	0.0500 (5)	
H11A	0.9932	0.1539	0.5471	0.060*	
C12	1.0100 (2)	0.0663 (4)	0.66754 (18)	0.0843 (9)	
H12A	0.9472	−0.0375	0.6614	0.126*	
H12B	1.0133	0.1446	0.7182	0.126*	
H12C	1.0796	−0.0153	0.6667	0.126*	
C13	1.09104 (19)	0.4058 (5)	0.60623 (16)	0.0752 (7)	
H13A	1.0797	0.5089	0.5614	0.113*	
H13B	1.1617	0.3271	0.6065	0.113*	
H13C	1.0931	0.4880	0.6560	0.113*	

### Atomic displacement parameters (Å^2^)

**Table:**

	*U*^11^	*U*^22^	*U*^33^	*U*^12^	*U*^13^	*U*^23^
O2	0.0286 (6)	0.0262 (6)	0.0431 (7)	−0.0024 (4)	−0.0035 (5)	−0.0012 (4)
O1	0.0390 (7)	0.0385 (7)	0.0323 (6)	0.0007 (5)	0.0031 (5)	0.0073 (5)
N1	0.0381 (8)	0.0242 (6)	0.0349 (8)	0.0044 (5)	0.0053 (6)	0.0016 (5)
O3	0.0401 (8)	0.0400 (8)	0.0817 (10)	−0.0055 (5)	0.0068 (7)	−0.0124 (7)
C1	0.0247 (8)	0.0269 (8)	0.0353 (9)	−0.0018 (6)	0.0021 (6)	0.0021 (6)
C8	0.0247 (8)	0.0271 (8)	0.0317 (8)	−0.0010 (6)	0.0044 (6)	0.0004 (6)
C9	0.0290 (8)	0.0312 (8)	0.0352 (8)	0.0019 (6)	0.0025 (6)	−0.0032 (6)
C10	0.0325 (9)	0.0390 (9)	0.0334 (9)	−0.0020 (7)	0.0074 (7)	−0.0007 (7)
C7	0.0252 (8)	0.0283 (8)	0.0332 (8)	−0.0003 (6)	0.0041 (6)	0.0025 (6)
C2	0.0263 (8)	0.0253 (8)	0.0300 (8)	−0.0016 (6)	0.0002 (6)	−0.0015 (6)
C3	0.0324 (9)	0.0277 (8)	0.0360 (9)	0.0022 (6)	0.0034 (7)	0.0036 (6)
C6	0.0346 (9)	0.0301 (9)	0.0415 (9)	0.0009 (6)	0.0085 (7)	−0.0054 (7)
C4	0.0377 (10)	0.0429 (10)	0.0333 (9)	−0.0001 (7)	0.0022 (7)	0.0066 (7)
C5	0.0340 (9)	0.0512 (11)	0.0298 (8)	−0.0023 (7)	0.0071 (6)	−0.0045 (7)
C11	0.0313 (10)	0.0610 (12)	0.0566 (12)	0.0022 (8)	0.0033 (8)	−0.0117 (10)
C12	0.0569 (15)	0.0593 (14)	0.127 (2)	0.0053 (11)	−0.0164 (14)	0.0254 (15)
C13	0.0335 (12)	0.1033 (19)	0.0886 (18)	−0.0095 (12)	0.0084 (11)	0.0082 (15)

### Geometric parameters (Å, °)

**Table:**

O2—C2	1.4356 (16)	C3—C4	1.395 (2)
O2—H2A	0.8200	C3—H3A	0.9300
O1—C1	1.2270 (18)	C6—C5	1.399 (2)
N1—C1	1.355 (2)	C6—H6A	0.9300
N1—C7	1.4144 (19)	C4—C5	1.375 (3)
N1—H1A	0.8600	C4—H4A	0.9300
O3—C10	1.204 (2)	C5—H5A	0.9300
C1—C2	1.557 (2)	C11—C13	1.522 (3)
C8—C3	1.378 (2)	C11—C12	1.527 (3)
C8—C7	1.393 (2)	C11—H11A	0.9800
C8—C2	1.510 (2)	C12—H12A	0.9600
C9—C10	1.513 (2)	C12—H12B	0.9600
C9—C2	1.518 (2)	C12—H12C	0.9600
C9—H9A	0.9700	C13—H13A	0.9600
C9—H9B	0.9700	C13—H13B	0.9600
C10—C11	1.511 (2)	C13—H13C	0.9600
C7—C6	1.379 (2)		
			
C2—O2—H2A	109.5	C8—C3—H3A	120.6
C1—N1—C7	111.85 (12)	C4—C3—H3A	120.6
C1—N1—H1A	124.1	C7—C6—C5	117.29 (15)
C7—N1—H1A	124.1	C7—C6—H6A	121.4
O1—C1—N1	126.39 (14)	C5—C6—H6A	121.4
O1—C1—C2	125.40 (14)	C5—C4—C3	120.42 (15)
N1—C1—C2	108.04 (13)	C5—C4—H4A	119.8
C3—C8—C7	120.23 (14)	C3—C4—H4A	119.8
C3—C8—C2	130.26 (14)	C4—C5—C6	121.53 (15)
C7—C8—C2	109.47 (12)	C4—C5—H5A	119.2
C10—C9—C2	114.65 (13)	C6—C5—H5A	119.2
C10—C9—H9A	108.6	C13—C11—C10	111.69 (16)
C2—C9—H9A	108.6	C13—C11—C12	111.02 (18)
C10—C9—H9B	108.6	C10—C11—C12	109.35 (17)
C2—C9—H9B	108.6	C13—C11—H11A	108.2
H9A—C9—H9B	107.6	C10—C11—H11A	108.2
O3—C10—C11	122.77 (15)	C12—C11—H11A	108.2
O3—C10—C9	120.45 (15)	C11—C12—H12A	109.5
C11—C10—C9	116.77 (14)	C11—C12—H12B	109.5
C6—C7—C8	121.75 (14)	H12A—C12—H12B	109.5
C6—C7—N1	129.26 (14)	C11—C12—H12C	109.5
C8—C7—N1	108.99 (13)	H12A—C12—H12C	109.5
O2—C2—C8	112.10 (12)	H12B—C12—H12C	109.5
O2—C2—C9	105.10 (11)	C11—C13—H13A	109.5
C8—C2—C9	115.95 (12)	C11—C13—H13B	109.5
O2—C2—C1	108.68 (11)	H13A—C13—H13B	109.5
C8—C2—C1	101.33 (11)	C11—C13—H13C	109.5
C9—C2—C1	113.69 (13)	H13A—C13—H13C	109.5
C8—C3—C4	118.76 (15)	H13B—C13—H13C	109.5

### Hydrogen-bond geometry (Å, °)

**Table:**

*D*—H···*A*	*D*—H	H···*A*	*D*···*A*	*D*—H···*A*
N1—H1A···O2^i^	0.86	2.22	3.0049 (18)	151
O2—H2A···O1^ii^	0.82	2.00	2.8220 (17)	175

Symmetry codes: (i) *x*, *y*+1, *z*; (ii) −*x*+1, −*y*+1, −*z*+1.

## References

[bb1] Brandenburg, K. (1999). *DIAMOND* Crystal Impact GbR, Bonn, Germany.

[bb2] Bruker (2002). *SMART* and *SAINT for WNT/2000* Bruker AXS Inc., Madison, Wisconsin, USA.

[bb3] Farrugia, L. J. (1997). *J. Appl. Cryst.* **30**, 565.

[bb4] Farrugia, L. J. (1999). *J. Appl. Cryst.* **32**, 837–838.

[bb5] Garden, S. J., da Silva, R. B. & Pinto, A. C. (2002). *Tetrahedron*, **58**, 8399–8412.

[bb6] Glover, V., Halket, J. M., Watkins, P. J., Clow, A., Goodwin, B. L. & Sandler, M. (1988). *J. Neurochem.* **51**, 656–659. 10.1111/j.1471-4159.1988.tb01089.x 3392550

[bb7] Li, Y. M., Zhang, Z. K., Jia, Y. T., Shen, Y. M., He, H. P., Fang, R. X., Chen, X. Y. & Hao, X. J. (2008). *Plant Biotechnol. J.* **6**, 301–308. 10.1111/j.1467-7652.2008.00322.x 18266823

[bb8] Marti, C. & Carreira, E. M. (2003). *Eur. J. Org. Chem.* pp. 2209–2219.

[bb9] Pandeya, S. N., Sriram, D., Nath, G. & DeClercq, E. (2000). *Eur. J. Med. Chem.* **35**, 249–255. 10.1016/s0223-5234(00)00125-2 10758286

[bb13] Sheldrick, G. M. (2005). *SADABS* University of Göttingen, Germany.

[bb10] Sheldrick, G. M. (2008). *Acta Cryst.* A**64**, 112–122. 10.1107/S0108767307043930 18156677

[bb11] Sun, L., Tran, N., Tang, F., App, H., Hirth, P., McMahon, G. & Tang, C. (1998). *J. Med. Chem.* **41**, 2588–2603. 10.1021/jm980123i 9651163

[bb12] Teitz, Y., Ronen, D., Vansover, A., Stematsky, T. & Riggs, J. L. (1994). *Antivir. Res.* **24**, 305–314. 10.1016/0166-3542(94)90077-9 7993075

